# LINC00612 enhances the proliferation and invasion ability of bladder cancer cells as ceRNA by sponging miR-590 to elevate expression of PHF14

**DOI:** 10.1186/s13046-019-1149-4

**Published:** 2019-04-02

**Authors:** Liying Miao, Hong Yue Liu, Cuixing Zhou, Xiaozhou He

**Affiliations:** 1 Department of Hemodialysis, The Third Affiliated Hospital of Soochow University, Changzhou Shi, China; 2 Department of Pharmacy, Fudan University Shanghai Cancer Center, Shanghai, China; 3 Department of Urology, The Third Affiliated Hospital of Soochow University, Changzhou, Jiangsu Province China; 4 Department of Oncology, Shanghai Medical College, Fudan University, Shanghai, China

**Keywords:** Bladder Cancer, LINC00612, ceRNA, EMT

## Abstract

**Background:**

Bladder cancer (BC) is a common type of cancer that involves tumors of the urinary system and poses a serious threat to human health. Long noncoding RNAs (lncRNAs) have emerged as crucial biomarkers and regulators in many cancers. Novel lncRNA biomarkers in BC urgently need to be investigated in regard to its function and regulatory mechanisms.

**Methods:**

Identification of differentially expressed lncRNAs in BC tissue was performed via microarray analysis. To investigate the biological functions of LINC00612, loss-of-function and gain-of-function experiments were performed in vitro and in vivo. Bioinformatics analysis, dual-luciferase reporter assays, AGO2-RIP assays, RNA pull-down assays, real-time quantitative PCR (RT-qPCR) arrays, fluorescence in situ hybridization assays, and western blot assays were conducted to explore the underlying mechanisms of competitive endogenous RNAs (ceRNAs).

**Results:**

*LINC00612* was upregulated in BC tissues and cell lines. Functionally, downregulation of *LINC00612* inhibited cell proliferation and invasion in vitro and in vivo, whereas overexpression of *LINC00612* resulted in the opposite effects. Bioinformatics analysis and luciferase assays revealed that *miR-590* was a direct target of *LINC0061*, which was validated by dual-luciferase reporter assays, AGO2-RIP assays, RNA pull-down assays, RT-qPCR arrays, and rescue experiments. Additionally, *miR-590* was shown to directly target the PHD finger protein 14 (*PHF14*) gene. *LNIC00612* modulated the expression of E-cadherin and vimentin by competitively sponging *miR-590* to elevate the expression of *PHF14*, thus affecting BC cellular epithelial-mesenchymal transition (EMT).

**Conclusions:**

Our results indicate that *LINC00612* enhances the proliferation and invasion ability of BC cells by sponging *miR-590* to upregulate *PHF14* expression and promote BC cellular EMT, suggesting that *LINC00612* may act as a potential biomarker and therapeutic target for BC.

## Introduction

Bladder cancer is a common type of cancer involving tumors of the urinary system, has the highest published incidence involving malignant urinary system tumors, and poses a serious threat to human health. According to data released by the US Department of Health, an estimated 76,960 patients were diagnosed with bladder cancer and 16,390 died of complications in 2016 [1]. The most common histopathological type of bladder cancer is transitional cell carcinoma (TCC), which accounts for more than 90% of bladder cancers, followed by squamous cell carcinoma, adenocarcinoma, and undifferentiated carcinoma [2]. Bladder cancer is characterized by multifocality and relapse. Most patients have non-muscle invasive bladder cancer at initial diagnosis. After active surgery and bladder perfusion therapy, relapse, infiltration and drug resistance can occur, which can threaten human health and socio-economic development [3]. The lack of specificity and sensitivity of technologies in the early diagnosis of bladder cancer, as well as the high rates of postoperative recurrence and malignant transformation following surgery, such as bladder tumor resections, are the major problems in bladder cancer diagnosis and treatment. Therefore, identification of new, highly sensitive, specific and cost-effective bladder cancer markers is needed to improve the early diagnosis rate of bladder tumors, which will have important clinical significance for ultimately improving the prognoses of patients.

LncRNAs are non-coding RNAs that are more than 200 nucleotides in length and that affect regulatory gene expression. LncRNAs lack a complete open reading frame and do not have a protein coding function. LncRNAs were first discovered by Okazaki et al. [4]. By using mouse DNA transcripts. Up to 4–9% of the sequences in the mammalian genome sequence can produce lncRNAs. LncRNAs were originally thought to be the “dark matter” or “noise” of genomic transcription and to have no biological function [5]. Later studies demonstrated that lncRNAs are involved in many important cellular functions, such as X chromosome silencing, genomic imprinting, chromatin modification, and transcriptional activation or inhibition, and can promote changes in molecular function in related signaling pathways, as well as alter cell life activities [6–8]. LncRNA plays a key role in the initiation, development and metastasis of bladder cancer. At present, *UCN-1*, *PVT-1*, *MALAT1*, *SPRY4-IT1*, *PANDAR*, *H19* and other lncRNAs closely related to bladder cancer have been identified. These lncRNAs affect important biological roles, such as proliferation, apoptosis, migration and invasion of bladder cancer, and also participate in disease progression and outcomes by regulating epigenetic modifications and key cell signaling transduction pathways. In this study, gene chip screening technology was used to discover lncRNAs related to the occurrence and development of bladder cancer and to identify their functions and regulation mechanisms to promote a new understanding of the pathogenesis of bladder cancer and to guide clinical treatment.

## Methods

### Microarray profiling

TRIzol Reagent (Invitrogen, Carlsbad, CA) was used to extract total RNA which was then purified by a RNeasy Mini Kit (Qiagen, Valencia, CA). Differentially expressed lncRNAs in BC and normal adjacent tissues were screened by the LncRNA microarray expression profiling based on the criteria of log2 (fold change) > 1.5 and adjusted *P* < 0.01. Manufacturer’s standard protocols were strictly followed. Briefly, cDNA was synthesized, labeled and purified. lncRNA microarray chips was hybridized by Cyanine-3-CTP labeled cRNA. Then after washing, samples were analyzed on the lncRNAs microarray. The differentially expressed genes were calculated and clustered by R program.

### Tissue samples

Resected BC and normal adjacent tissues were collected from The Third Affiliated Hospital of Soochow University from Jan 2013 to Jan 2016. There were 13 BC samples and 8 normal adjacent tissue samples. All tissues were directly stored in liquid nitrogen at − 80 °C. Informed consent was obtained from each participant. The use of human clinical tissues was approved by the Institutional Human Experiment and Ethics Committee of The Third Affiliated Hospital of Soochow University. The Declaration of Helsinki was strictly followed during experiments.

### Cell line culture

Cell lines were purchased from American Type Culture Collection (ATCC, Manassas, VA, USA) including BC cell lines (5637, UMUC3 and T24), human bladder epithelium immortalized cells (SV-HUC-1) and human embryonic kidney cell line (HEK-293). Cells were maintained in modified RPMI-1640 medium, supplemented with 10% fetal bovine serum (FBS) including 100 μg/L penicillin and 100 μg/L streptomycin. All cell lines were grown with 5% CO_2_ at 37 °C.

### Real-time quantitative polymerase chain reaction (RT-qPCR)

TRIzol reagent (Invitrogen, Carlsbad, CA, USA) was used to isolated total RNA from tissues and cells according to the manufacturer’s instructions. Moloney Leukemia Virus Reverse Transcriptase Kit (Promega, Madison, WI, USA) was then performed to reverse transcribe total RNA(1 μg) to cDNA. Target primers were amplified by SYBR Green Mix (Promega). Sequences of the primers are listed in Table 1. All primers were synthesized by Shanghai Tingzhou Biological Engineering Co., Ltd. The *miR-590* level was performed using TaqMan MicroRNA Assays Kit (Applied Biosystems, Carlsbad, CA, USA) according to the manufacturers’ instructions. All results were calculated and expressed as 2^-ΔΔCt^. GAPDH was used as endogenous control for *LINC00612* and *PHF14* and U6 for *miR-590*. Triplicate is required for each experiment.

**Table 1:** Primer used in this study

ID	Sequences
LINC00612 forward	5′-GGCAGAGCCATGTGTTGGATA-3′
LINC00612 reverse	5′-GTGCTCCCTAATGGCTCACA-3′
PHF14 forward	5′-GCAACTTGCAAGGGAACTGG-3’
PHF14 reverse	5′-AAGAGGTTTCCGGGATTGCC-3’
GAPDH forward	5′-TGAACGGGAAGCTCACTGG-3’
GAPDH reverse	5′-TCCACCACCCTGTTGCTGTA-3’
U6 forward	5′-CTCGCTTCGGCAGCACA-3’
U6 reverse	5′-AACGCTTCACGAATTTGCGT-3’

### RNA isolation of nuclear and cytoplasmic fractions

The Nuclear/Cytoplasmic Isolation Kit (Biovision) was applied to isolate and collect cytosolic and nuclear fractions. RNA levels of *LINC00612*, *RNU6–1*(nuclear control transcript) and GAPDH (cytoplasmic control transcript) were analyzed by RT-qPCR.

### In situ hybridization (ISH)

Cells were seeded onto poly-L-lysine-treated glass slides for 24 h after trypsinization harvest and then fixed in methanol at − 20 °C for 5 min. The ISH assays were performed as previously described [9]. A locked nucleic acid probe with complementarity to a section of *LINC00612* (5′- TATCGAACTTTCTAGATCGGTGCAC-3′ custom LNA detection probe, Exiqon) was labeled with digoxigenin antibody (Roche, 11,093,274, 1:1000) and synthesized. The intensity and the extent of staining were evaluated by 2 pathologists who were blinded to the experiment.

### Fluorescence in situ hybridization (FISH)

Five thousand six hundred thirty-seven and T24 cells were fixed in 4% PFA for 15 min. Then, 0.5% TritonX-100 was used to permeabilize the cells for 15 min at 4 °C. Digoxigenin (DIG)-labeled *LINC00612* probe or control probe mix were performed to incubate cells for 4 h at 55 °C. After 2 × saline-sodium citrate briefly washing for 5 min (5–6 times), signal was detected by Horseradish peroxidase (HRP)-conjugated anti-DIG secondary antibodies (Jackson, West Grove, PA, USA). Olympus confocal laser scanning microscope was applied for image obtaining. DAPI was used to counterstain nuclear.

### IHC

IHC staining was performed as previously described [9]. Briefly, the tumor tissues were cut into 4-mm-thick sections, dewaxed in xylene and rehydrated in a graded series of alcohols. Antigen was retrieved by heating the tissue sections at 100 °C for 30 min in EDTA solution (1 mM, pH 9.0). Cooled tissue sections were immersed in 0.3% hydrogen peroxide solution for 15 min to block endogenous peroxidase activity, rinsed with phosphate-buffered saline (PBS) for 5 min and blocked with 3% BSA solution at room temperature for 30 min. Subsequently, the sections were incubated with mouse monoclonal antibody against human *PHF14* (1:200) at 4 °C overnight, followed by incubation with HRP-conjugated goat anti-rabbit secondary antibody. Diaminobenzene was used as the chromogen, and hematoxylin was used as the nuclear counterstain.

### Lentivirus production and cell transfection

The pLVX-IRES-Puro vector for *LINC00612* overexpression and lentivirus-containing short hairpin RNA (shRNA) targeting *LINC00612* (top strand: Top Strand 5′-CACCGGTAGATGACAGATTAGATACCGAAGTATCTAATCTGTCATCTACC-3′; bottom strand: 5′-AAAAGGTAGATGACAGATTAGATACTTCGGTATCTAATCTGTCATCTACC-3′) were purchased from Genelily BioTech Co., Ltd., (Shanghai, China). The cells were selected by puromycin (2 μg/mL) for 2 weeks at 48 h after transfection. Cell lines with stable *LINC00612* silence or overexpression was then constructed. RT-qPCR was performed to verify the transfection efficiency. The *miR-590 mimic*, *miR-590 inhibitor*, and negative control (NC) oligonucleotides were obtained from Tingzhou Biological Engineering Co., Ltd. (Shanghai, China). Abovementioned oligonucleotides and plasmids were transfected by using Lipofectamine 3000 (Invitrogen). The manufacturer’s instructions were strictly followed.

### Cell counting Kit-8 (CCK8) and Colony formation assay

Cells (2 × 10^4^ cells/ml) were seeded onto 96-well plates (100 μL/well) and then placed in an incubator with 5% CO_2_ at 37 °C for 24 h. After the cells were cultured for 5 days, 10 μl of CCK8 solution was added to each well. The absorbance values at a wavelength of 450 nm were measured to evaluate cell viability. For colony formation assay, BC cells colon spheres were generated as previously described [10]. Briefly, cells (500 cells/well) were seeded in to 6-well plates for 24 h. Cells were incubated for 2 weeks, then fixed in methanol and stained with 0.1% crystal violet. Quantity One software (Bio-Rad, Hercules, CA, USA) was used to count colonies. Triplicate is required for each experiment.

### Transwell assay

Transwell chambers (8-μm pore size; Corning Costar, Cambridge, MA, USA) was applied to measure cell invasion ability. Instruction was strictly followed. Cells were suspended in serum-free RPMI-1640 medium, then seeded into the upper chamber. Serum (20%) was supplemented into the lower chamber that containing RPMI-1640 medium which was regarded as a chemoattractant. After 48 h incubation, the filters were fixed in methanol and stained with 0.1% crystal violet. The upper faces of the filters were gently abraded. Cells migrated across the lower faces of filters were imaged and counted under the microscope. Triplicate is required for each experiment.

### Western blot analysis

Western blot analyses were performed according to standard protocols as previously described [11].

Anti-*E-cadherin*, Anti-*N-cadherin*, Anti-vimentin and Anti-*PHF14* were purchased from Sigma.

### Luciferase reporter assays

The reporter vector pmirGLO-LINC00612-wt was formed by cloning *LINC00612* cDNA which contains predictive binding site of *miR-590* into the pmirGLO Dual-Luciferase miRNA Target Expression Vector (Promega). The vector pmirGLO-LINC00612-Mut was inserted by the mutant *LINC00612* that containing point mutations of the *miR-590* seed region binding site. HEK-293FT cells were cultured and co-transfected with pmirGLO-LINC00612–3′-UTR vectors including wild-type or mutant fragments, *miR-590* and *miR-NC*. Likewise, wild-type and mutant *PHF14* 3′-UTR fragments were cloned into the pmirGLO vector. The *miR-590* or *miR-NC* was co-transfected with *PHF14-wt* or *PHF14-Mut* vector into HEK-293FT cells using Lipofectamine 3000 (Invitrogen). The Dual Luciferase Reporter Assay System (Promega) was applied at 48 h after transfection according to the manufacturer’s instructions. Triplicate is required for each experiment.

### RNA immunoprecipitation

The EZMagna RIP Kit (Millipore) was applied according to the manufacturer’s protocol. Complete RNA immunoprecipitation (RIP) lysis buffer was used to lyse BC cells. Magnetic beads conjugated with Anti-Argonaute 2 (AGO2) or control anti-IgG antibody were performed in incubation of cell extract. The cell extract was incubated for 6 h at 4 °C. Then RT-qPCR analysis for purified RNA was conducted as removal of proteins of the beads had been done.

### RNA pull down assay

Briefly, T24 cells were transfected with the 3’end biotinylated *miR-590* or *miR-590-mut* or several candidate miRNAs for 24 h at a final concentration of 20 nmol/L. Then, the cells were incubated in the cell lysate with streptavidin-coated magnetic beads (Ambion, Life Technologies). The biotin-coupled RNA complex was pulled down and analysis of the abundance of *LINC00612* in bound fractions was then conducted by RT-qPCR. The pull-down assay was performed as previously described [12].

### Xenograft tumor model

Xenograft tumor model was performed in BALB/c-nude mice (4–5 weeks of age) which were purchased from Shanghai SLAC Laboratory Animal Co., Ltd., China. The experimental procedures were approved by the Institutional Animal Care and Use Committee of our institution. Tumor growth was monitored every 5 days; tumor volumes were estimated by length and width. One month later, the mice were sacrificed, then tumors were excised and weighed.

### Abdominal metastasis model

After anesthetization, a left lateral flank incision was operated on the mice. The spleen was then exteriorized. About 100 μl of Hank’s balanced salt solution that containing T24-Luc-vector and T24-Luc-*siLINC00612* cells (8 × 10 [6]) were injected into the spleen parenchyma by 25-gauge needle. The IVIS bioluminescence imaging system (Caliper Life Sciences) was performed to collect bioluminescence images after day 28. All experiments were approved by the relevant guidelines of The Third Affiliated Hospital of Soochow University.

### Statistical analysis

SPSS 22.0 statistical software package and GraphPad Prism 7.0 were applied for statistical analyses.

All data are represented as mean ± standard deviation (SD). To compare two or more groups, the Student’s t-test or one-way analysis of variance (ANOVA) were performed for differences analysis. Differences were considered statistically significant when *P* < 0.05.

## Results

### *LINC00612* was up-regulated in BC tissues and cell lines

**Fig. 1:**
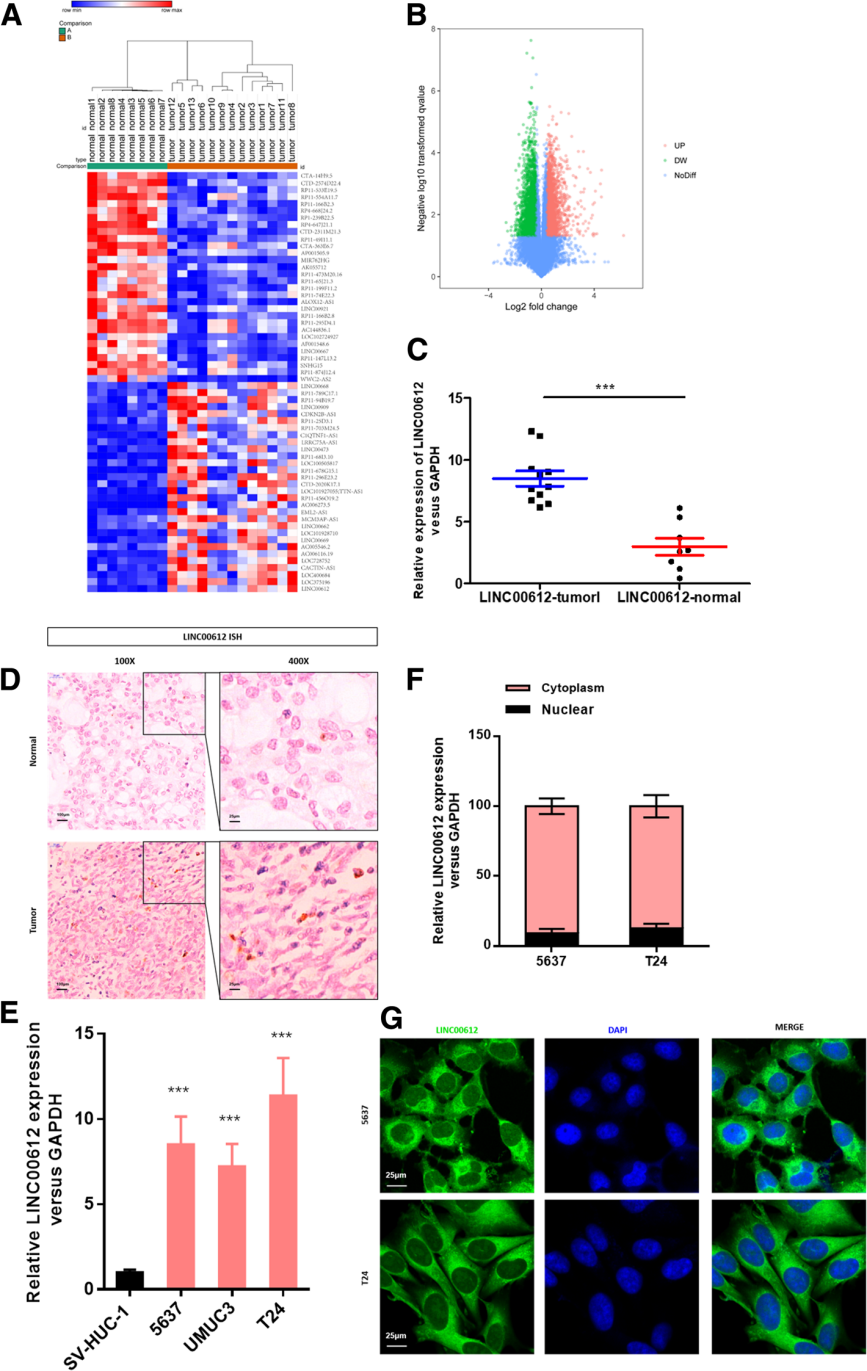
*LINC00612* is upregulated in bladder cancer (BC) tissues and cell lines. **a** Heat map with hierarchical clustering of the top 60 differentially expressed lncRNAs between BC samples and normal samples (> 1.5-fold; *P* < 0.05). **b** LncRNAs (with fold change > 1.5 and *P* < 0.05) plotted as a volcano plot. **c** RT-qPCR was performed to validate *LINC00612* expression in BC samples and normal samples (*n* = 13 vs. *n* = 8, respectively). ***P < 0.001. **d** In situ hybridization detection of *LINC00612* in BC tissue and normal tissue (× 100 and × 400). **e** RT-qPCR was performed to measure the relative expression of *LINC00612* in BC cell lines (5637, UMUC3 and T24) and human bladder epithelium immortalized cells (SV-HUC-1). The majority of *LINC00612* was located in the cytoplasm, according to the nuclear mass separation assay (**f**) and fluorescence in situ hybridization (**g**)

A total of 60 lncRNAs (fold change > 1.5, Padj < 0.01) differentially expressed in BC tissues were screened (Fig. 1a & b). As shown in the heat map, *LINC00612* was significantly up-regulated in tumor tissues compared with that in normal tissues (Fig. 1a). The results of ISH and RT-RT-qPCR revealed that the expression of *LINC00612* was higher in tumor tissues than that in normal tissues (Fig. 1c & d). According to the results of RT-qPCR, the expression of *LINC00612* in BC cell lines (5637, UMUC3 and T24) was significantly elevated compared with that in human bladder epithelium immortalized cells (SV-HUC-1) (Fig. 1e), and there was a statistically significant difference (*P* < 0.05). The subcellular localization of *LINC00612* in BC cell lines was determined using the nuclear mass separation assay and FISH, and it was found that *LINC00612* was mainly located in the cytoplasm (Fig. 1f & g).

### Ablation of *LINC00612* inhibited cell viability and invasion of BC cells

**Fig. 2:**
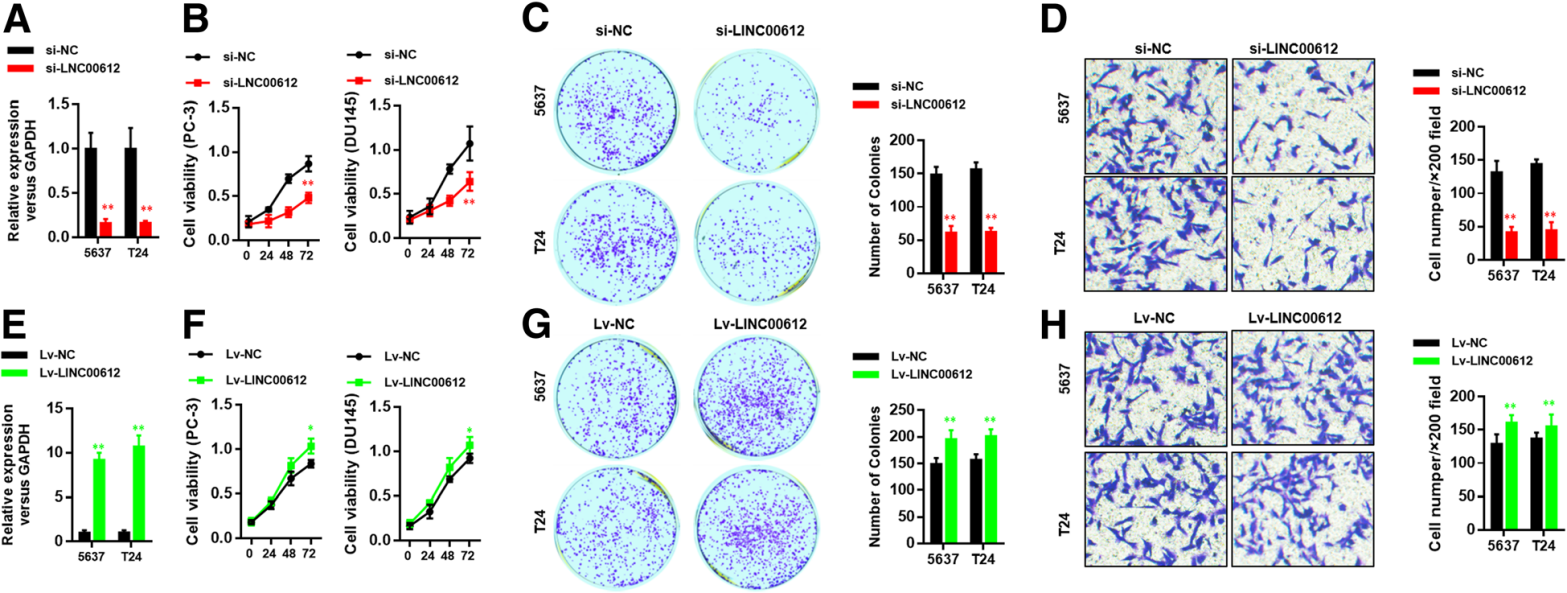
*LINC00612* promotes cell proliferation and invasion of BC cells in vitro. **a** Efficiency of *LINC00612* expression in *LINC00612* downregulated 5673 and T24 cells was evaluated via RT-qPCR. **P < 0.01. **b** Cell viability was measured via CCK8 assays. **P < 0.01. **c**, **d** Representative results of colony formation and transwell assays of 5673 and T24 cells after *shLINC00612* or *shNC* transfection. **e** Efficiency of *LINC00612* expression in *LINC00612* overexpressed 5673 and T24 cells was evaluated via RT-qPCR. **P < 0.001. **f** Cell viability was measured via CCK8 assays. *P < 0.05. **g**, **h** Representative results of colony formation and transwell assays of 5673 and T24 cells after *Lv-LINC00612* or *Lv-NC* transfection. *N* = 3 independent experiments

Loss-of-function experiments was used to determine whether *LINC00612* influences BC cell proliferation and invasion. RT-qPCR was applied to verify knockdown efficiency of *LINC00612*. After transfection with the vector containing shLINC00612, the expression of *LINC00612* in 5637 and T24 cells was obviously reduced compared with that in empty vector group (Fig. 2a). To evaluate cell viability, we subsequently performed CCK8 and colony formation assays. The results showed that cell growth and colony formation were strongly inhibited by ablation of *LINC00612* (Fig. 2b & c). Also, knockdown of *LINC00612* weakened the cell invasive and migratory capacity (Fig. 2d). Consistently, when we transfected the vector that containing *LINC00612* overexpression plasmid into BC cells, the expression of *LINC00612* in 5637 and T24 cells was significantly increased compared with that in empty vector group (Fig. 2e). Cell growth and colony formation were thus enhanced (Fig. 2f & g) as well as the cell invasive and migratory capacity (Fig. 2h). The differences were statistically significant (*P* < 0.05).

### In vivo verification of influence of *LINC00612* on cell proliferation and invasion ability

**Fig. 3:**
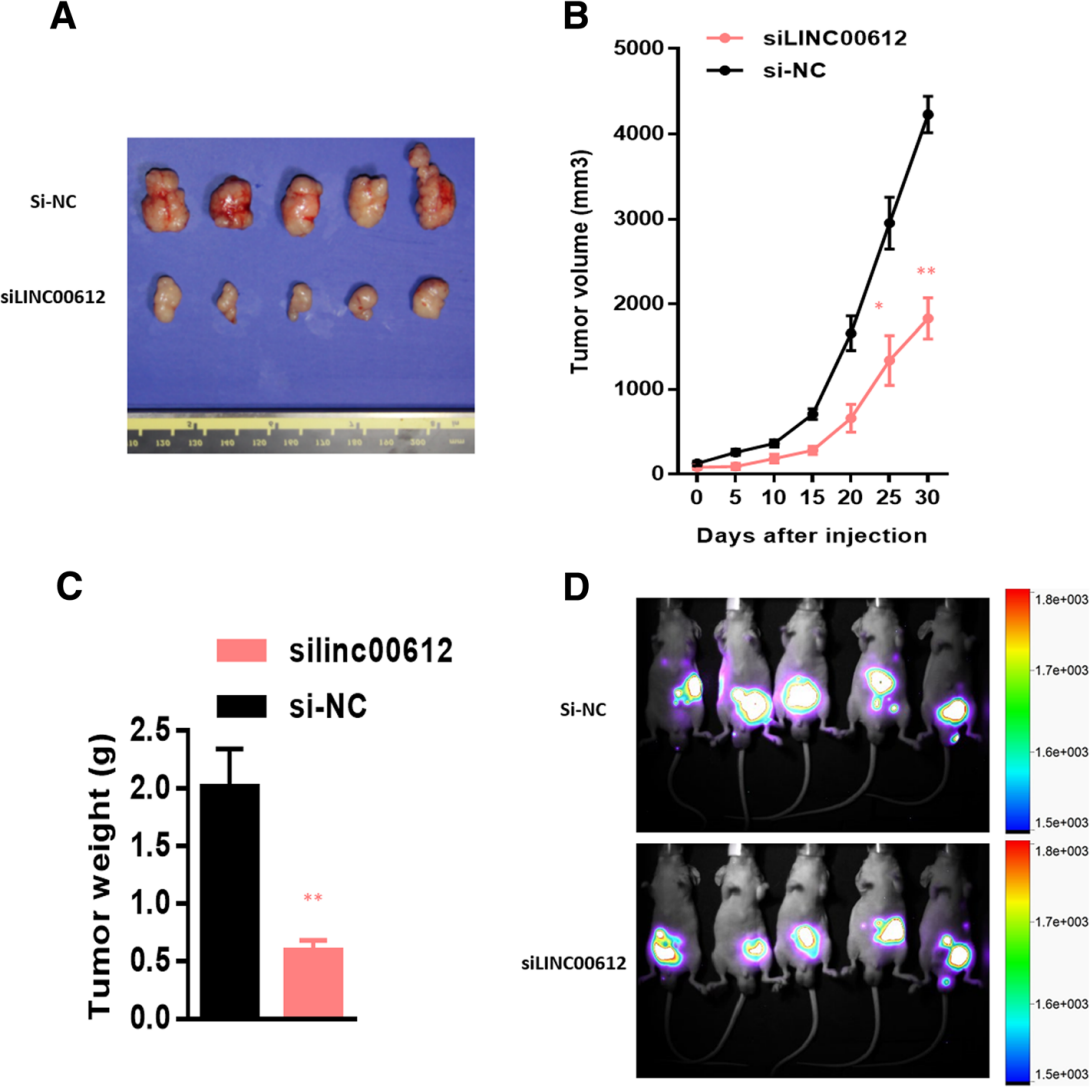
Ablation of *LINC00612* inhibits proliferation and metastasis in vivo. **a** Nude mice were subcutaneously injected with T24 cells transfected with the *shLINC00612* vector and control vector. Tumors were removed after 1 month. Both tumor volume (**b**) and tumor weight (**c**) were analyzed between the *shLINC00612* and control groups. **d** Bioluminescent imaging of *shLINC00612* and luciferase expressing control T24 cells transplanted in nude mice. **P < 0.01

To evaluate the influence of *LINC00612* on proliferation and invasion ability, BALB/c-nude mice were injected subcutaneously with the T24 cells with stable down-regulation of *LINC00612* and corresponding control cells. As expected, the transplanted tumor with the down-regulation of *LINC00612* had a smaller volume and lower weight than that in control group (Fig. 3a, b & c). To investigate the effect of *LINC00612* on metastases, an in vivo abdominal metastasis model was performed. We injected approximately 1 × 10^7^ *LINC00612* down-regulated and control T24 cells into spleen of BALB/c-nude mice and used bioluminescence imaging to evaluate abdominal metastasis. One month after injection, bioluminescence imaging confirmed that *LINC00612* ablation significantly inhibited metastasis (Fig. 3d). The differences were statistically significant (*P* < 0.05).

### *LINC00612* played a role in BC as an endogenous competitive RNA of *miR-590* and regulated target gene *PHF14* of *miR-590*

**Fig. 4:**
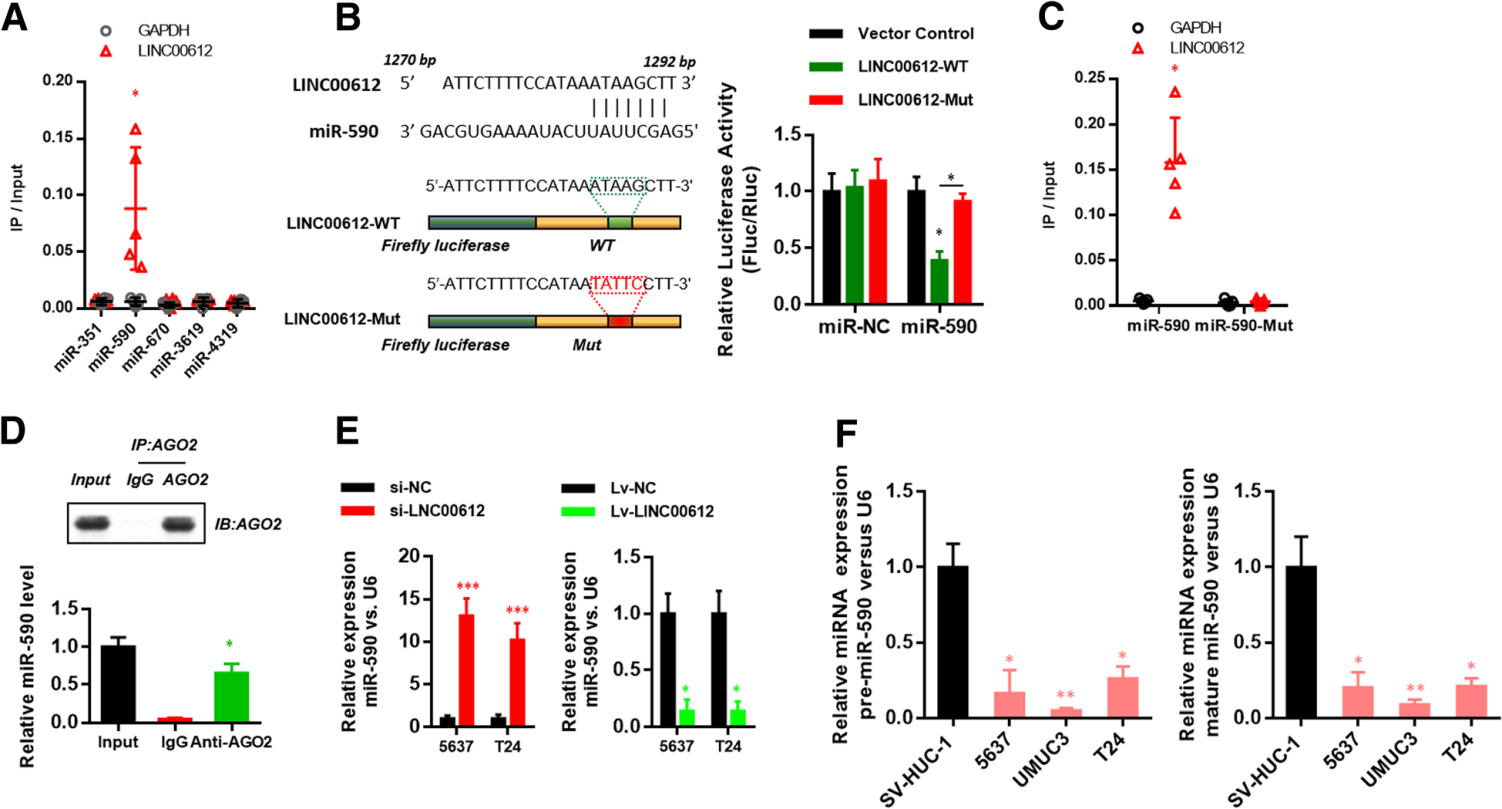
*LINC00612* served as a molecular sponge for *miR-590*. **a** Biotinylated miRNA were transfected into T24 cells. RT-qPCR was performed to quantify the RNA levels of *LINC00612* and *GAPDH*. A scatter plot showing the relative ratios of the input of IP. *P < 0.05. **b** A schematic diagram showing the putative *miR-590* binding sites with the *LINC00612*. The sequences of wild-type *LINC00612* and mutant *LINC00612* are also listed. Luciferase reporter gene assays were performed to measure the luciferase activity in T24 cells. *P < 0.01. **c** Biotinylated *miR-590* or its mutant (*miR-590-mut*) was transfected into T24 cells. RT-qPCR was performed to quantify the RNA levels of *LINC00612* and *GAPDH*. A scatter plot showed the relative ratios of the input of IP. *P < 0.01. **d** Anti-AGO2 RIP assays were used in T24 cells to determine *LINC00612* and *miR-590* RNA enrichment in IP complexes. Anti-IgG was used as a control. *P < 0.01. **e** Relative expression of *miR-590* in BC cells that were transfected with the *Lv-LINC00612* vector/*Lv-NC* vector and *shLINC00612* vector/*shNC* vector were measured via RT-qPCR. **f** RT-qPCR was performed to measure the relative expression of *pre-miR590* and *mature-miR590* in BC cell lines (5637, UMUC3, and T24) and human bladder epithelium immortalized cells (SV-HUC-1). **P < 0.01. *N* = 3 independent experiments

The competitive endogenous RNA (ceRNA) is a well-known regulatory mechanism of lncRNA. LncRNA sponges a variety of miRNAs to inhibit its expression and reduce the regulatory effect on target mRNA. The target recognition sequence of *LINC00612* miRNA was analyzed via bioinformatics (RegRNA 2.0 http://regrna2.mbc.nctu.edu.tw/detection.html; Lncrnadb http://www.lncrnadb.org; miRcode http://www.mircode.org/), and it was found that several miRNAss had a complementary sequence to *LINC00612*. RNA pull-down was performed to screening the most relevant target miRNA. The result indicated that *LINC00612* was more enriched in the *miR-590* compared with other miRNAs (Fig. 4a). To prove the finding, *LINC00612* cDNA was cloned into the luciferase gene (pGL3-PVT1–214-wt) and co-transfected with *miR-590* or *miR-NC.* The results revealed that the luciferase activity in *miR-590* group was significantly reduced compared with that in *miR-NC* group. At the same time, the *miR-590* binding site was mutated, and the pGL3-PVT1–214-mut vector was produced. The results showed that the vector after mutation had no significant influence on the luciferase activity in *miR-590* group (Fig. 4b). The results of RNA pull-down assay manifested that *LINC00612* was more enriched in the wild-type *miR-590* compared with that in the mutant-type *miR-590* with broken *LINC00612* binding site (Fig. 4c). RNA induced silencing complexes (RISCs) is formed by miRNA ribonucleoprotein complexes (miRNPs) which is present in anti-Ago2 immunoprecipitates. Therefore, anti-Ago2 immunoprecipitates contain miRNAs and their interacting RNA-components [13–15]. RIP assay was performed using anti-AGO2 in the T24 extract, and it was found that *LINC00612* and *miR-590* were enriched preferentially in miRNPs containing AGO2 compared with anti-IgG immunoprecipitates (Fig. 4d). The RT-qPCR results showed that down-regulation of *LINC00612* could cause the increase in *miR-590* expression, while overexpression of *LINC00612* in BC cells could lead to the down-regulation of *miR-590* expression (Fig. 4e). RT-qPCR results displayed that the expression of pre-*miR-590* and mature *miR-590* in BC cell lines (5637, UMUC3 and T24) was significantly reduced compared with that in human bladder epithelium immortalized cells (SV-HUC-1) (Fig. 4f).

**Fig. 5:**
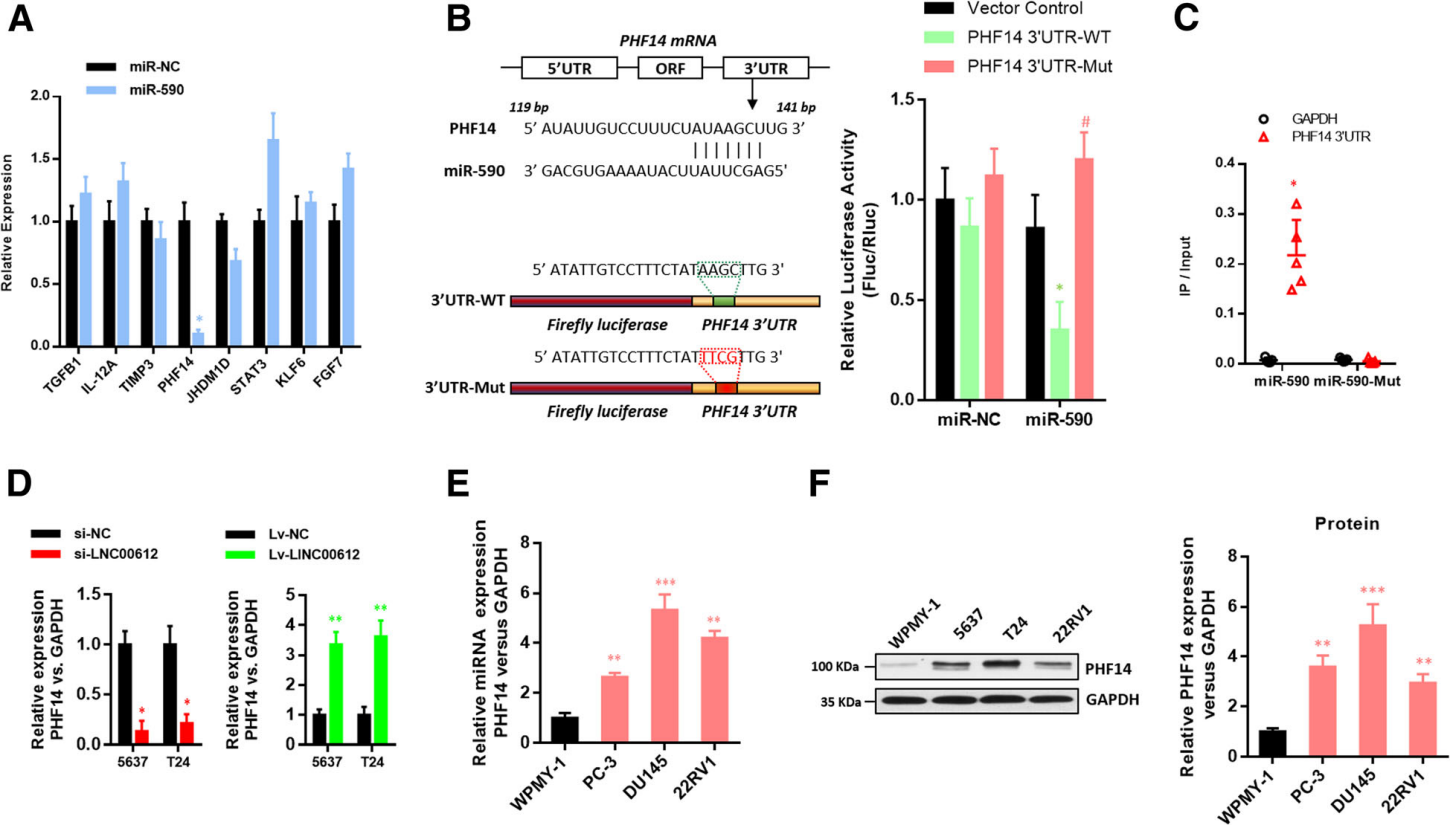
*PHF14* was identified as a direct target of *miR-590* in BC cells. **a** *miR-590* and *miR-NC* were transfected into T24 cells. RT-qPCR was performed to quantify the RNA levels of several candidates. *P < 0.01. **b** Schematic diagram showing the predicted *miR-590* binding sites within the 3’UTR of *PHF14*. The sequences of wild-type and mutant 3’UTR of *PHF14* are also listed. Luciferase reporter gene assays were performed to measure the luciferase activity in T24 cells. *P < 0.01. **c** Biotinylated *miR-590* or its mutant (*miR-590-mut*) was transfected into T24 cells. RT-qPCR was performed to quantify the RNA levels of the 3’UTR of *PHF14* and *GAPDH*. Scatter plot showing the relative ratios of the input of IP. *P < 0.01. **d** The relative expressions of *PHF14* in BC cells transfected with the *Lv-LINC00162* vector/*Lv-NC* vector and *shLINC00612* vector/*shNC* vector were measured via RT-qPCR. **P < 0.01. **e**, **f** RT-qPCR and western blots were performed to determine the relative expression of *PHF14* in BC cell lines (5637, UMUC3, and T24) and human bladder epithelium immortalized cells (SV-HUC-1). ***P < 0.001. *N* = 3 independent experiments

**Fig. 6:**
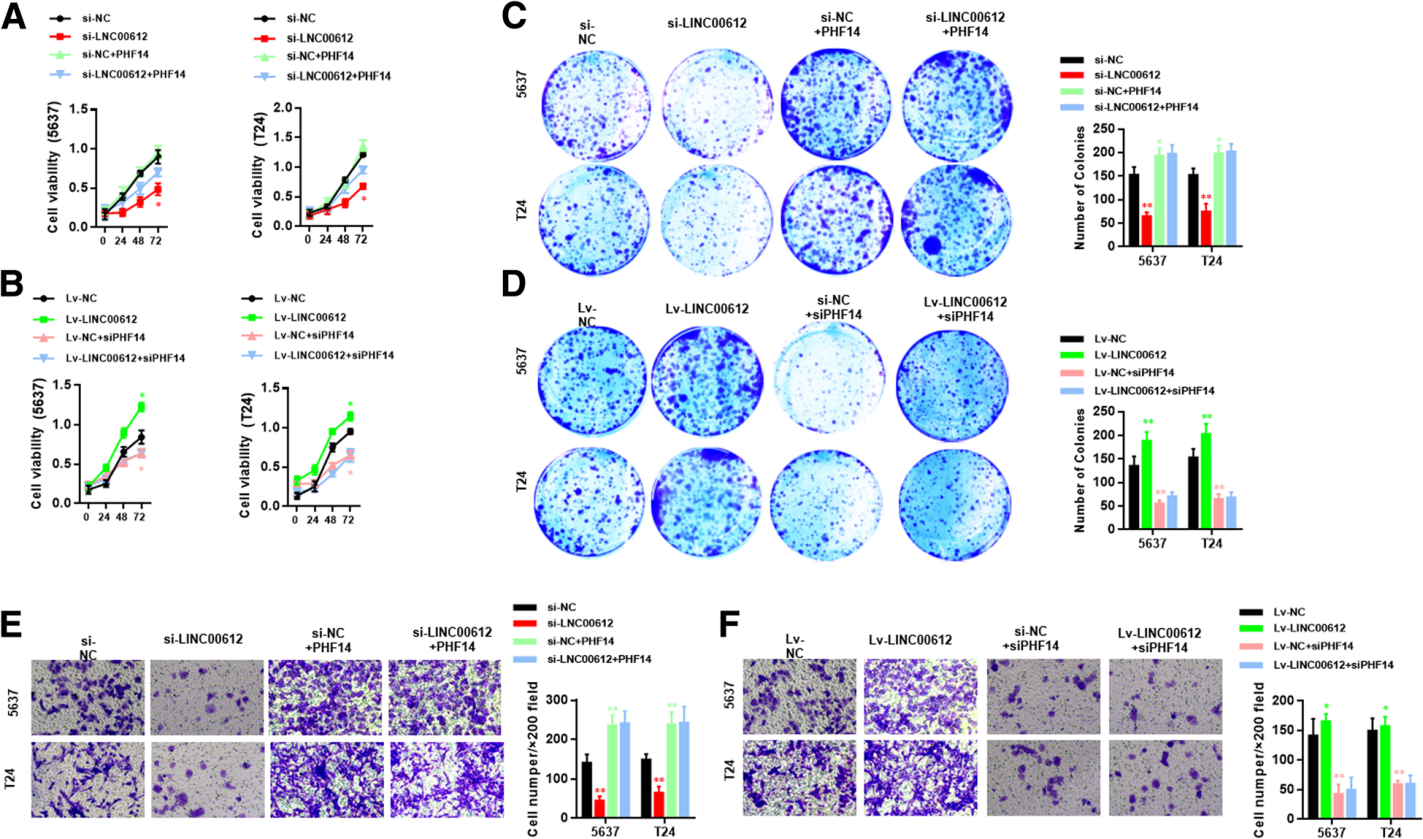
CCK-8 assays (**a**, **b**) and colony formation assays (**c**, **d**) were performed to measure the cell viability and a transwell assay (**e**, **f**) was performed to measure the cell invasion ability in 5637 and T24 cells after transfection with *shNC*/*Lv-NC*, *shLINC00612*/*Lv-LINC00612*, *shNC + Lv-PHF14*/*Lv-NC + shPHF14* and *shLINC00612 + Lv-PHF14*/*Lv-LINC00612 + shPHF14*. **P < 0.01. *N* = 3 independent experiments

According to the prediction via bioinformatics (Targetscan 7.2 http://www.targetscan.org/vert_72/; miRDB http://www.mirdb.org/; miRTarBase http://mirtarbase.mbc.nctu.edu.tw/php/index.php), there were binding sites in several genes’ 3′-UTR with *miR-590*. Then RT-qPCR was used to screening. Finally, *PHF14* was identified as target gene (Fig. 5a). Then the luciferase assay confirmed that the 3′-UTR of wild-type *PHF14* could significantly lower the luciferase activity in *miR-590* group without significant influence on the luciferase activity in *miR-NC* group. The 3′-UTR of mutant-type *PHF14* had no obvious influence on the luciferase activity in *miR-590* group (Fig. 5b). RNA pull-down assay confirmed that *PHF14* 3′-UTR was more enriched in the wild-type *miR-590* compared with that in the mutant-type *miR-590* with broken *PHF14* 3′-UTR binding site (Fig. 5c). Whether *PHF14* could be regulated by the expression of *LINC00612* was verified then. According to the RT-qPCR results, the overexpression or down-regulation of *LINC00612* could increase or decrease the mRNA expression in *PHF14* (Fig. 5d). RT-qPCR and western blotting results verified that the expression of *PHF14* in BC cell lines (5637, UMUC3 and T24) was significantly increased compared with that in human bladder epithelium immortalized cells (SV-HUC-1) (Fig. 5e & f). Moreover, the down-regulation of *LINC00612* expression in BC cells could reverse the promotion on cell viability (Fig. 6a & c) and invasive and migratory capacity (Fig. 6e) caused by *PHF14* overexpression. Accordingly, the overexpression of *LINC00612* could reverse inhibition on cell viability (Fig. 6b & d) and invasive and migratory capacity caused by *PHF14* knock-down (Fig. 6f).

### *LINC00612/miR-590/PHF14 axis* regulated BC cell epithelial-mesenchymal transition (EMT)

**Fig. 7:**
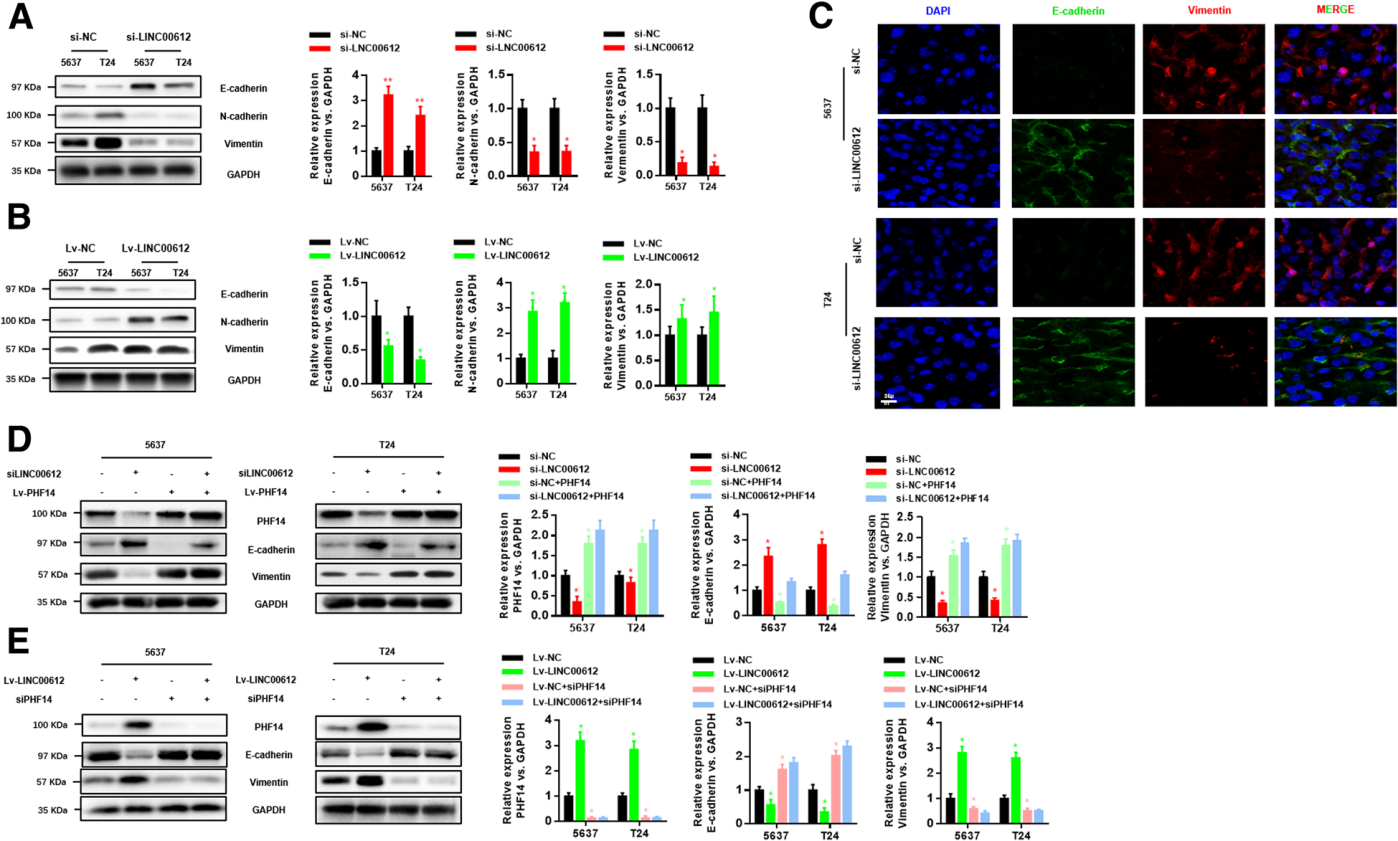
Western blot analysis was performed to evaluate the expression of E-cadherin, N-cadherin, and vimentin proteins in 5637 and T24 cells after *LINC00612* knockdown (**a**) and overexpression (**b**). **c** Representative images of subcellular localization via FISH. The expression levels of E-cadherin (green) and vimentin (red) were identified. Nuclei were counterstained with the use of DAPI (blue). **d**, **e** Western blot analysis was performed to examine the expression of PHF14, E-cadherin and vimentin proteins in 5637 and T24 cells after transfection with *shNC*/*Lv-NC*, *shLINC00612*/*Lv-LINC00612*, *shNC + Lv-PHF14*/*Lv-NC + shPHF14*, and *shLINC00612 + Lv-PHF14*/*Lv-LINC00612 + shPHF14*. **P < 0.01. *N* = 3 independent experiments

EMT is one of major cause that epithelial-derived malignant cells gained increased migration and invasion ability. To evaluate the effect on EMT of *LINC00612*, western blotting assay was performed. It could be seen in the result that knock-down of *LINC00612* enhanced the expression of the epithelial marker *E-cadherin* and weakened the expression of *N-cadherin* and the mesenchymal marker *vimentin* (Fig. 7a). Correspondingly, overexpression of *LINC00612* inhibited the expression of *E-cadherin* and promoted the expression of *N-cadherin* and *vimentin* (Fig. 7b). This conclusion also had been manifested by FISH (Fig. 7c). We further explored the role of *LINC00612/miR-590/PHF14* pathway that affect EMT. The ablation of *LINC00612* could reverse the promotion on expression of vimentin and inhibition on expression of *E-cadherin* caused by *PHF14* overexpression (Fig. 7d). The similar results were also observed in *Lv-LINC00612 + siPHF14* group (Fig. 7e). The results suggested that *LINC00612/miR-590/PHF14 axis* is a novel regulator in BC progression and metastasis.

### In vivo verification of impact of LINC00612/miR-590/PHF14 axis

**Fig. 8:**
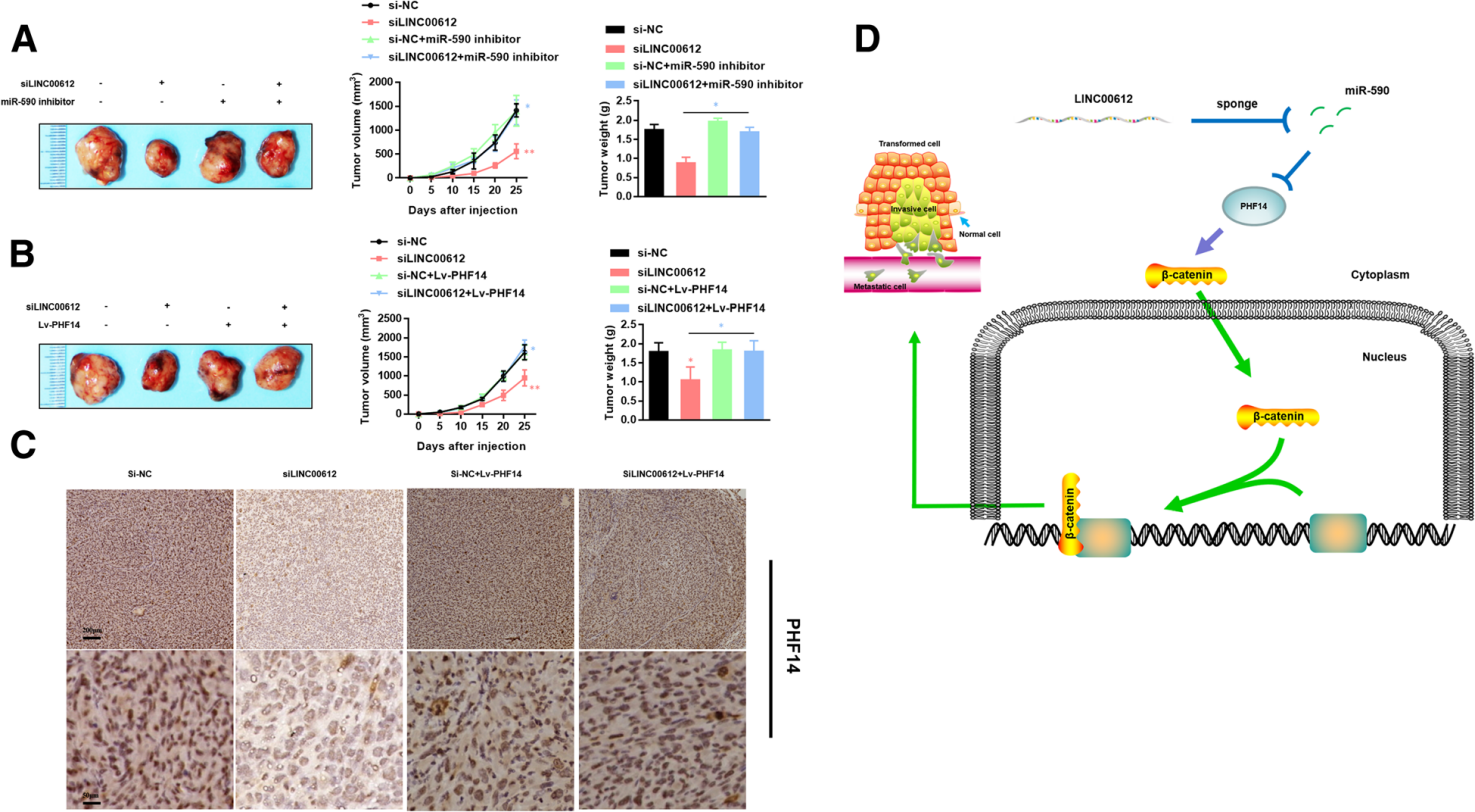
Tumors were collected from nude mice injected with T24 cells transfected with *siNC*, *siLINC00612*, *miR-590* inhibitor + *siNC*, *miR-590* inhibitor + *siLINC00612* (**a**), and *siNC*, *siLINC00612*, *Lv-PHF14* + *siNC*, *Lv-PHF14* + *siLINC00612* (**b**). Tumor volume was analyzed every 5 days. Tumor weight was measured 30 days after the tumor transplantation. The differences in tumor volume and mass between the co-transfecting group (*siLINC00612* + *miR-590* inhibitor and *siLINC00612* + *Lv-PHF14*) and the negative control group were not statistically significant. *P < 0.05, compared with the NC group. **c** IHC detection of PHF14 in paraffin-embedded tissue sections. **d** Possible molecular mechanisms of the *LINC00612*/*miR-590*/*PHF14* axis in bladder cancer: *LINC00612* competitively binds to *miR-590*, which could directly combine with *PHF14*. *PHF14* could enhance the proliferation and invasion ability of bladder cancer cells by activating the WNT pathway via the promotion of migration of β-catenin from the cytoplasm into the nucleus. *LINC00612* weakens the inhibiting effect of *miR-590* on *PHF14*

In nude mice xenografts, the tumor volume and mass could be significantly affected by modulation of *miR-590* and *PHF14* in T24 which subcutaneously injected to nude mice (*P* < 0.05). The transplanted tumor with the transfection of *siLINC00612* had a smaller volume and lower weight than that in control group. However, the tumor suppression effect in vivo caused by *siLINC00612* was neglected when co-transfected with *siLINC00612* + *miR-590 inhibitor* or *siLINC00612 + Lv-PHF14*. The differences on tumor volume and mass between co-transfecting group and their negative control group were not statistically significant. The IHC results showed that *PHF14* was less expressed in *siLINC00612* tumor tissue than those in control, *Lv-PHF14* and *siLINC00612 + Lv-PHF14* tumor tissues. (Fig. 8). All these results suggested that *LINC00612/miR-590/PHF14 axis* could eventually modulate the proliferation in BC cells.

## Discussion

In recent years, several lncRNAs related to the development of bladder cancer have been identified. Wang et al. [16] reported that exogenous expression of urothelial cancer associated 1 (*UCA1*) in the bladder transitional cell carcinoma cell line BLS-211 enhanced the proliferation, migration, invasion and drug resistance of bladder cancer cells. Wu et al. [17] reported that *UCA1* can regulate the proliferation of bladder cancer cell lines through the PI3KAKT-mTOR signaling pathway. Furthermore, they also confirmed that the transcription factor CCAAT/enhancer-binding protein (C/EBP)-α can affect the expression level of *UCA1*. *UCA1* inhibits the growth of bladder cancer cells by inhibiting the expression of the transcription factor C/EBP-α. In addition, a number of studies have shown that *UCA1* expression can be detected in tumors of bladder cancer patients, as well as in their urine, and exhibits tumor tissue specificity [18–20]. *UCA1* exhibits high sensitivity, strong specificity, and stable experimental results with respect to bladder cancer. Chen et al. [21] used microarray analysis to screen differentially expressed lncRNAs and demonstrated that the expression level of the lncRNA *n336928* in bladder cancer tissues was significantly higher than that in paracancerous tissue and that expression of the lncRNA *n336928* was positively correlated not only with the stage and grade of bladder tumors but also with age, gender, smoking status, tumor size, and tumor number. Survival analyses revealed that the 5-year survival rate of patients in the high lncRNA *n336928* expression group was significantly lower than that in the low expression group. Liu et al. [22] reported that expression of *SPRY4-IT1* in bladder cancer tissues and bladder cancer cell lines was higher than that in paracancerous normal tissues and normal bladder epithelial cells. Downregulation of *SPRY4-IT1* expression by siRNA interference significantly inhibited the proliferation and migration of bladder cancer cells and promoted apoptosis of bladder cancer cells [23]. To date, little is known about the role of *LINC00612* in tumors. In this study, we found that *LINC00612* is significantly upregulated in bladder cancer tissues and cell lines. Further studies confirmed that *LINC00612* could promote tumor proliferation and invasion in vivo and in vitro, suggesting that *LINC00612* may be a potential target for observation and treatment of bladder cancer.

Researchers at Harvard University proposed the ceRNA hypothesis in 2011, arguing that there is a pattern of interactions between miRNA and mRNA. The hypothesis further states that various types of RNA molecules (including mRNA, lncRNA, etc.) can regulate each other by competitively inhibiting miRNAs via common microRNA response elements (MRE) [24]. The stability and transcription of cytoplasmic lncRNA can be modified by trapping miRNAs, thereby altering signaling pathways. In this study, we found that *LINC00612* was primarily localized in the membrane, which indicated that the ceRNA mechanism may exist. Subsequently, bioinformatic predictions, luciferase reporter gene experiments, RIP experiments, and RNA pull-down experiments revealed that *LINC00612* directly binds and sponges *miR-590*. *miR-590* has been shown to play an important role in a variety of cancers, and its function has been described as being carcinostatic in breast cancer, osteosarcoma and lung cancer [25–28]. However, some studies reached different conclusions. These studies suggested that *miR-590* could promote tumor cell proliferation and enhance tolerance to radiotherapy [6–8]. The effect of *miR-590* in bladder cancer remains unclear. In this study, we showed that *miR-590* could be sponged by *LINC00612* and therefore counter the carcinogenic effect of *LINC00612*, which provides a basis for confirming the carcinostatic effect of *miR-590* in bladder cancer. Agostino [29] suggested that *miR-590* can be specifically adsorbed by the lncRNA *MIR205HG* in head and neck squamous cell carcinomas, thereby leading to uncontrolled tumor cell proliferation. This ceRNA regulation mechanism was similar to our findings.

EMT is a biological process in which epithelial-derived malignant cells transform into mesenchymal cells with increased migration and invasion ability [30]. The characteristic changes in EMT include the loss of polarity of epithelial cells, degradation of intercellular junctions, changes in cell morphology due to the reorganization of cytoskeletal structures, and downregulation of epithelial gene expression accompanied by upregulation of mesenchymal gene expression. These changes provide the cell with a greater ability to migrate, invade and degrade the extracellular matrix [31]. During EMT, cells lose epithelial marker factors, such as E-cadherin, while mesenchymal markers increase, such as vimentin, N-cadherin, and fibronectin, and related transcription factors, including Twist, Snail, and Zeb families, are activated. The results of this study conclude that *LINC00612* promotes EMT in BC cells by inhibiting the expression of the epithelial marker E-cadherin and by enhancing the expression of the mesenchymal marker vimentin, thus increasing the proliferation and invasion of BC cells. This regulation was competitively adjusted by *miR-590*, according to the ceRNA mechanism. *miR-590* has been previously reported as being an EMT inhibitory miRNA [32], which is in accordance with our findings. Furthermore, we demonstrated that *miR-590* could directly bind to downstream *PHF14* at the 3’UTR. *PHF14* is involved in several signaling pathway, including the classical TGF-β signaling pathway [33]. Several researchers have demonstrated that *PHF14* is overexpressed in biliary tract cancer and lung cancer and may also be involved in tumorigenesis [34, 35]. To measure whether the regulation of the *LINC00612*/*miR-590/PHF14* axis modulated cellular EMT and thus modulated the proliferation and invasion of BC cells, rescue experiments in vitro and in vivo were conducted. When *LINC00612*/*miR-590* and *LINC00612*/*shPHF14* (*shLINC00612*/*PHF14*) were cotransfected into BC cells, the alterations in cellular EMT, cell proliferation and invasion were restored. These results confirmed that the *LINC00612*/*miR-590*/*PHF14* axis had a substantial effect on BC cellular EMT and might be a crucial modulator of cell proliferation and invasion in BC cells. At present, the function and regulation of *LINC00612* in other tumors remains unclear. Meanwhile, the regulation of the *LINC00612*/*miR-590*/*PHF14* axis in bladder cancer requires confirmation in large-scale clinical research studies, which will be the main aim of a future study.

## Conclusion

In summary, our results suggest that the expression of *LINC00612* is elevated in bladder cancer and can promote tumor cell proliferation and invasion in bladder cancer, as confirmed by in vivo and in vitro experiments. Furthermore, we suggest a possible regulatory mechanism through the sponging of *miR-590* by *LINC00612*, leading to upregulation of *PHF14*, promoting tumor cellular EMT and enhancing the proliferation and invasion of bladder cancer cells. *LINC00612* may be a novel bladder cancer marker and a potential therapeutic target.

## Abbreviations

BC: Bladder Cancer
ceRNAs: competitive endogenous RNAs
EMT: epithelial-mesenchymal transition
FISH: Fluorescence in situ hybridization
ISH: In situ hybridization
PHF14: PHD finger protein 14
RT-qPCR: Real-Time quantitative PCR arrays
