# Correction to: LINC00612 enhances the proliferation and invasion ability of bladder cancer cells as ceRNA by sponging miR-590 to elevate expression of PHF14

**DOI:** 10.1186/s13046-022-02326-0

**Published:** 2022-03-15

**Authors:** Liying Miao, Hong Yue Liu, Cuixing Zhou, Xiaozhou He

**Affiliations:** 1 Department of Hemodialysis, The Third Affiliated Hospital of Soochow University, Changzhou Shi, China; 2 Department of Pharmacy, Fudan University Shanghai Cancer Center, Shanghai, China; 3 Department of Urology, The Third Affiliated Hospital of Soochow University, Changzhou, Jiangsu Province China; 4 Department of Oncology, Shanghai Medical College, Fudan University, Shanghai, China

**Correction to: J Exp Clin Cancer Res 38, 143 (2019)**

**https://doi.org/10.1186/s13046-019-1149-4**

Following publication of the original article [1], the authors identified minor errors in Fig. 2; specifically:

- Fig. 2d: Incorrect images used for transwell assays of 5673 and T24 cells after *shLINC00612* or *shNC* transfection (first column); correct images now used

The corrected figure is given here. The correction does not have any effect on the final conclusions of the paper.

**Fig. 2:**
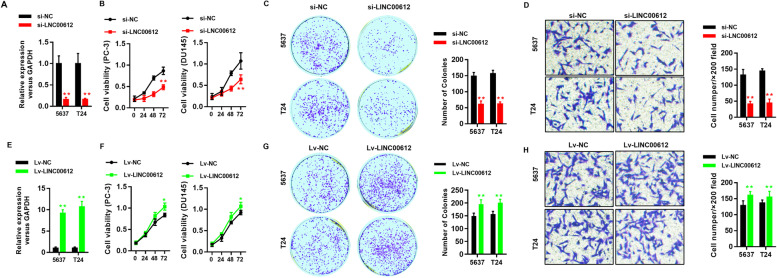
*LINC00612* promotes cell proliferation and invasion of BC cells in vitro. **a** Efficiency of *LINC00612* expression in *LINC00612* downregulated 5673 and T24 cells was evaluated via RT-qPCR. **P < 0.01. **b** Cell viability was measured via CCK8 assays. **P < 0.01. **c**, **d** Representative results of colony formation and transwell assays of 5673 and T24 cells after *shLINC00612* or *shNC* transfection. **e** Efficiency of *LINC00612* expression in *LINC00612* overexpressed 5673 and T24 cells was evaluated via RT-qPCR. **P < 0.001. **f** Cell viability was measured via CCK8 assays. *P < 0.05. **g**, **h** Representative results of colony formation and transwell assays of 5673 and T24 cells after *Lv-LINC00612* or *Lv-NC* transfection. *N* = 3 independent experiments
